# HIV/AIDS late presentation and its associated factors in China from 2010 to 2020: a systematic review and meta-analysis

**DOI:** 10.1186/s12981-021-00415-2

**Published:** 2021-12-11

**Authors:** Chengqing Sun, Jianjun Li, Xiaoyan Liu, Zhi Zhang, Tao Qiu, Haiyang Hu, You Wang, Gengfeng Fu

**Affiliations:** 1 School of Public Health, Nanjing Medical University, Nanjing, People’s Republic of China; 2 Jiangsu Provincial Center for Disease Control and Prevention, Jiangsu, People’s Republic of China; 3 Department of Radiology, Fourth Affiliated Hospitial Of Nanjing Medical University, Nanjing, Jiangsu People’s Republic of China

**Keywords:** China, HIV/AIDS care, Late presentation, Associated factors, Meta-analysis

## Abstract

**Background:**

Late presentation to HIV/AIDS care presents serious health concerns, like increased transmission and high healthcare costs, increased mortality, early development of opportunistic infection, increased risk of antiretroviral therapy drug resistance. Despite the effort to contain the HIV/AIDS epidemic, LP has remained an impediment to individual immune reconstitution and public health.

**Objective:**

This review aimed to estimate the prevalence and determine the factors associated with late presentation to HIV/AIDS care.

**Methods:**

We searched PubMed, Web of Science, China National Knowledge Infrastructure (CNKI), Chinese Wanfang, and Weipu database for articles published from 2010 to 2020. We utilized *I*^*2*^ statistics and *Q*-test to estimate heterogeneity between studies. Random-effects meta-analysis models were used to calculate the aggregate odds ratio of late presentation to HIV/AIDS care.

**Results:**

Of 9563 titles and abstracts retrieved, 189 were identified as potentially eligible and 39 fulfilled the inclusion criteria. The pooled prevalence of late presentation to HIV/AIDS care was 43.26%. The major risk factors were patients ≥ 50 years old (*OR* = 2.19, 95% CI: 1.85–2.58; *I*^*2*^ = 97.44%), married (*OR* = 1.50, 95% CI: 1.35–1.68; *I*^*2*^ = 96.58%), with heterosexual contact as risk factor for infection (*OR* = 1.91, 95% CI: 1.73–2.11; *I*^*2*^ = 90.74%) and diagnosed in medical institutions (*OR* = 2.35,95% CI: 2.11–2.62; *I*^*2*^ = 96.05%). In middle or low HIV prevalence areas, patients ≥ 50 years old (*P* = 0.01), married (*P* < 0.01) and diagnosed in medical institutions (*P* = 0.01) were more likely to be presented late than in high prevalence areas. From 2016–2020, the OR of patients who were married and diagnosed in medical facilities were significantly lower than before (*P* < 0.01).

**Conclusion:**

Patients ≥ 50 years old, married, with heterosexual contact as risk factor for infection, and diagnosed in medical institutions were risk factors of LP. Gender had no significant relationship with LP. In middle or low prevalence areas, patients who were ≥ 50 years old, married, and diagnosed in medical institutions were more likely to be presented late than in other areas. Married patients and those diagnosed in medical institutions after 2015 have a lower risk of LP than before.

**Supplementary Information:**

The online version contains supplementary material available at 10.1186/s12981-021-00415-2.

## Introduction

Late presentation (hereon in referred to as LP) to HIV care remains a challenge to HIV prevention and treatment in the world. In Guatemala from 2000 to 2015 [1], 81.1% of new diagnoses were considered late presentations. The prevalence of LP is estimated to range between 36.9% in Estonia to 64.2% in Poland in Europe during 2010–2016 [2]. In China, the percentage of patients with late HIV presentation ranged from 35.5 to 42.1% from 2010 to 2014 [3]. LP may lead to some grave consequences both for individuals and the society, such as increased mortality, development of opportunistic infection [4], increased risk of antiretroviral therapy (ART) drug resistance [5], high healthcare costs [6], and increased transmission because of unawareness of infection status [7]. Some comprehensive strategies, such as extensive study, free testing, and prompt treatment initiation have been taken [8, 9]. However, only 75.7% of people who live with HIV know their infectious status by the end of 2019 in China.

Improving early presentation is of great importance for AIDS prevention and care. Early presentation means early entry to HIV care. Study evidence showed that patients shtarting early ART could have a near-normal life expectancy, provided that they start treatment before their CD4 count decreases below 200 cells/µl [10]. Early ART can also limit the HIV reservoir size [11, 12]. And the smaller the HIV reservoir at treatment interruption, the better the post-treatment control [13]. To some degree, ART also can prevent sexual HIV transmission in both homosexual and heterosexual individuals [14].

To our knowledge, there is only two published systematic review and meta-analysis on HIV late presentation and its predictors [15, 16]. In China, there are no such publications. Therefore, it is necessary to comprehend the situation of LP to HIV care and propose an effective program to promote early presentation in China. This meta-analysis aimed to estimate the pooled prevalence of LP and determine the risk factors from 2010 to 2020 in China. We hope to provide evidence for comprehensive prevention and testing strategy.

## Methods

### Literature search strategy

We searched PubMed, Web of Science, China National Knowledge Infrastructure (CNKI), Chinese Wanfang, and Weipu databases for articles published in English and Chinese from 2010 to 2020. Using the following Boolean term to search the databases: (TS = “HIV/AIDS” OR TS = “human immunodeficiency virus” OR TS = “Acquired immunodeficiency syndrome”) AND (TS = “late presentation” OR TS = “late diagnosis” OR TS = “late testing” OR TS = “delay presentation” OR TS = “delay diagnosis” OR TS = “delay testing”) in Chinese and (TS = "HIV/AIDS") AND (TS = "late entry" OR TS = "Advanced HIV disease" OR TS = "late presentation" OR TS = "late Diagnosed") in English. We also retrieved the studies referenced in all included studies to obtain further related studies. Our analyses followed the 2009 Preferred Reporting Items for Systematic reviews and Meta-Analyses(PRISMA) statement.

### Inclusion and exclusion criteria

Articles included met all the following criteria: (1) observational studies, including cross-sectional, case–control, and cohort studies; (2) studies reported the prevalence and associated factors of LP to HIV care in China from 2010 to 2020; (3) sufficient data present to estimate the odds ratios (ORs) with 95% confidence intervals (CIs); (4) studies defined late presentation of HIV/AIDS according to the European definition or other definitions recognized appropriate. Articles were excluded based on at least one of the following: (1) studies that do not meet the criteria above; (2) studies absence of original data, such as the number of patient with different factors; (3) studies published based on the same or overlapping data; (4) review, case report or meeting report.

### Data extraction and quality assessment

The studies searched were managed by Endnote (version X9) and de-duplicated. The studies were first screened by title and abstracts independently by two investigators according to the inclusion and exclusion criteria. If there were any conflicts between the results, all three authors of this review would screen the full text to discuss and resolve it before reaching a consensus. Moreover, the references cited in all included studies were screened by two investigators independently. Information retrieved from each eligible study comprised of:- title, first author, publication year, study design, study period, study time, the definition of LP, geographical locations, source population, number of subjects in each category, the value of odds ratios(*OR*) and it's 95% CI. The Newcastle–Ottawa quality-assessment scale (NOS) was employed to assess the quality of those included studies. We evaluated the quality of evidence using the "grading of recommendations assessment, development, and evaluation" (GRADE) approach.

### Statistical analysis

We estimated heterogeneity between studies with *I*^*2*^ statistics and *Q*-test. Random-effect models were administered in testing significant heterogeneity (*P* < 0.10 or *I*^*2*^ ≥ 50% implied statistically significant heterogeneity). Otherwise, fixed-effect models were applied. Subgroup analysis focused on study region and period. The study regions were categorized into high prevalence regions (including Yunnan, Guangxi, Henan, Sichuan, Xinjiang, and Guangdong provinces) and others according to the proportion of HIV infected patients in the nationally reported cases [17]. χ^2^ test was employed to estimate the proportion difference between the subgroups.

Sensitivity analysis was employed to assess the sensitivity of each included study. Publication bias was measured using the Harbord test and funnel plots (*P* > 0.05 represented no publication bias). We conducted all statistical analysis with Stata 16.0 and SPSS 24.0.

## Results

### Study review and selection

**Fig. 1:**
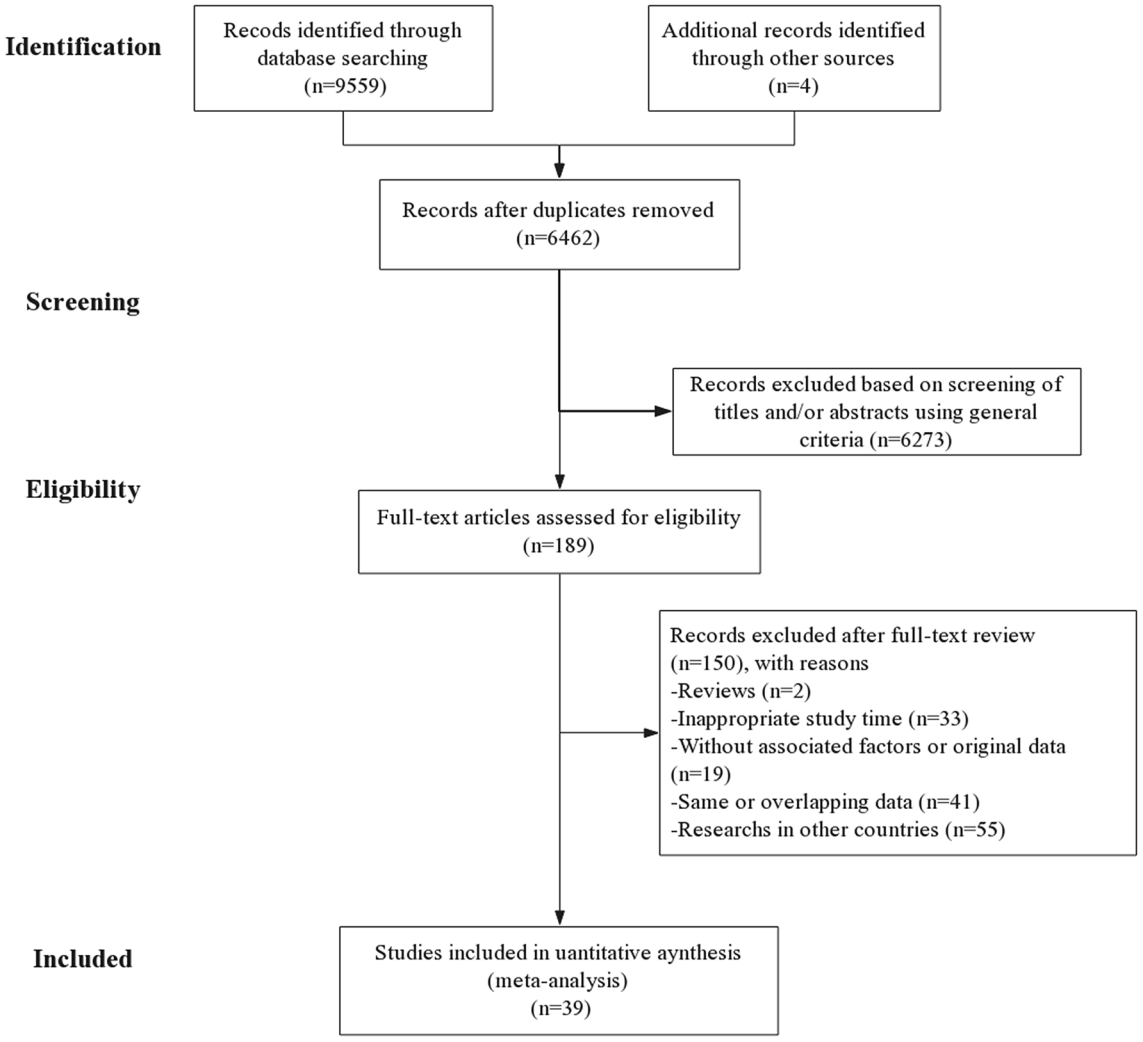
PRISMA 2009 Flow Diagram. Study identification and selection of the articles

A total of 4381 Chinese articles and 5182 English articles were obtained (CNKI 2624; Wanfang 1373; Weipu 384; PubMed 3931; Web of Science 1251). After the removal of duplicates, 6462(67.6%) remained. A total of 189 articles were eligible following titles and abstracts screening. After the full-text screening, 39 papers were finally included (36 Chinese and 3 English studies). The flowchart of studies identified by the search is in Fig. 1.

### General characteristic of the included studies

All of the 39 studies were case–control studies. According to the NOS assessment, the scores of all studies were ≥ 5, which denoted good quality. Included papers were from 2010 to 2020. The study regions covered 21 provinces or municipalities: Guangxi, Guangdong, Sichuan, Xinjiang, Yunnan, Jiangsu, Taiwan, Anhui, Beijing, Fujian, Gansu, Guizhou, Jiangxi, Liaoning, Shandong, Shaanxi, Shanxi, Tianjin, Zhejiang, Chongqing, and Hubei. We classified all study regions into high HIV prevalence or middle/low HIV prevalence groups based on HIV prevalence. Among these provinces and municipalities, Guangxi, Guangdong, Sichuan, Xinjiang, Yunnan are high HIV prevalence areas. Twenty-six (26) studies investigated the LP rate and evaluated the association between patients with age ≥ 50 and LP. The relationship between gender and LP was reported in 32 studies, and 38 publications researched the relationship between being married and LP. The number of studies that investigated the association of risk factor for infection and sample source with LP was 35. There are 12 articles with study time between 2010–2015, while four studies investigated the participants after 2015. The characteristics of the included publications are in Table 1.

**Table 1:** Publication characteristics of the included studies in this meta-analysis

First author (year)	Study design	Study period	Study region	No. of participants		
				Total(%)	Late presentation	Non-late presentation
				229,695(100.00)	105,953	123,742
Xi Hu [18]	Cross-sectional study	2012–2016	Guangxi Zhuang Autonomous Region	45,118(19.64)	31,663	13,455
Haiyang Hu [19]	Case–control	2011–2014	Jiangsu Province	491(0.21)	188	303
Hongbo Jiang [20]	Case–control	2018–2019	Guangdong Province	997(0.43)	400	597
Lin Jin [21]	Case–control	2011–2015	Anhui province	7073(3.08)	2949	4124
Ji Zeng [22]	Case–control	2013	Beijing City	2770(1.21)	582	2188
Yalan Huang [23]	Case–control	2011–2017	Quanzhou City, Fujian Province	2551(1.11)	901	1650
Jian Li [24]	Case–control	2013–2015	Gansu Province	1965(0.86)	524	1441
Ziming Lin [25]	Case–control	2010–2016	Guangdong Province	47,343(20.61)	19,624	27,719
Wenjie Cao [26]	Case–control	2014–2018	Guizhou Province	33,611(14.63)	10,495	23,116
Li Liu [27]	Case–control	2011–2015	Nanjing City, Jiangsu Province	3112(1.35)	963	2149
Liqiang Xu [28]	Case–control	2010–2015	Changshu City, Jiangsu Province	310(0.13)	120	190
Jinwei Li [29]	Case–control	2010–2015	Jingjiang City, Jiangsu Province	102(0.04)	36	66
Yao Qi [30]	Case–control	2011–2014	Yancheng City, Jiangsu Province	411(0.18)	148	263
Pengfei Bing [31]	Case–control	2012–2017	Suzhou City, Jiangsu Province	3605(1.57)	829	2776
Ping Liu [32]	Case–control	2013–2018	Zhangjiagang City, Jiangsu Province	401(0.17)	117	284
Lu Ye [33]	Case–control	2010–2017	Zhengjiang City, Jiangsu Province	972(0.42)	333	639
Qing Yang [34]	Case–control	2014–2018	Jiangxi province	11,557(5.03)	5227	6330
Dan Zhou [35]	Case–control	2015–2018	Liaogning Province	11,043(4.81)	3148	7895
Ying Wang [36]	Case–control	2014–2018	Heze City, Shandong Province	728(0.32)	252	476
Jianzhuo Li [37]	Case–control	2011–2016	Jinan City, Shandong Province	1365(0.59)	273	1092
Li Li [38]	Case–control	2012–2017	Linyi City, Shandong Province	887(0.39)	465	422
Hongmei Liang [39]	Case–control	2011–2016	Shanxi Province	5213(2.27)	1885	3328
Hailan Zhang [40]	Case–control	2011–2017	Xi’an City, Shaanxi Province	7427(3.23)	2088	5339
Zairan Duan [41]	Case–control	2012–2016	Hejiang County, Sichuan Province	693(0.30)	282	411
Yan Guo [42]	Case–control	2011–2015	Tianjin City	2922(1.27)	916	2006
Lirong Liu [43]	Case–control	2011–2015	Yining City, Xinjiang Uygur Autonomous Region	2449(1.07)	500	1949
Shunzhu Yin [44]	Case–control	2012–2018	Dali Bai Autonomous Prefecture, Yunnan Province	4648(2.02)	1467	3181
Lin Li [45]	Case–control	2015	Dehong Prefecture, Yunnan Province	942(0.41)	526	416
Zuokai Yang [46]	Case–control	2015–2017	Shaoxing City, Zhejiang Province	776(0.34)	202	574
Xiaohong Pan [47]	Case–control	2012	Zhejiang Province	1894(0.82)	500	1394
Yong Zhu [48]	Case–control	2012–2017	Rongchang District, Chongqing City	931(0.41)	442	489
Conghui Xu [49]	Case–control	2016	Shapingba District, Yubei District, Jiangjin district and Hechuan District of Chongqing City	1035(0.45)	349	686
Zhongrong Yang [50]	Case–control	2015–2017	Huzhou city, Zhejiang Province	757(0.33)	581	176
Qi Sun [51]	Case–control	2013–2019	Weihai City,Shandong Province	807(0.35)	526	281
Jie Ding [52]	Case–control	2010–2018	Wuhan City,Hubei Province	7783(3.39)	4815	2968
Jin Chen [53]	Case–control	2019	Xinjiang Uygur Autonomous Region	5489(2.39)	4723	766
Jiaxiang Chen [54]	Case–control	2010–2019	Jimei District, Xiamen City, Fujian Province	527(0.23)	368	159
Chenquan Qiu [55]	Case–control	2014–2019	Qujing City,Yunnan Province	7242(3.15)	5295	1947
Chunling Huang [56]	Case–control	2019	Suining City, Sichuan Province	1748(0.76)	1251	497

### Factors associated with LP

**Fig. 2:**
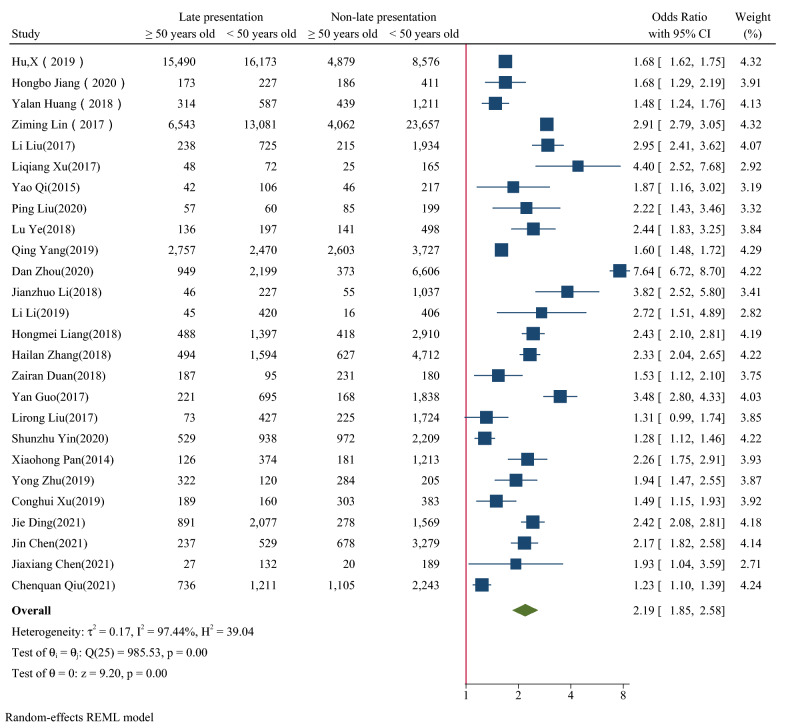
The forest plot of the association between age and late presentation. The midpoint and length of each segment indicated the OR and 95% confidence interval. The diamond shape revealed the pooled OR

**Fig. 3:**
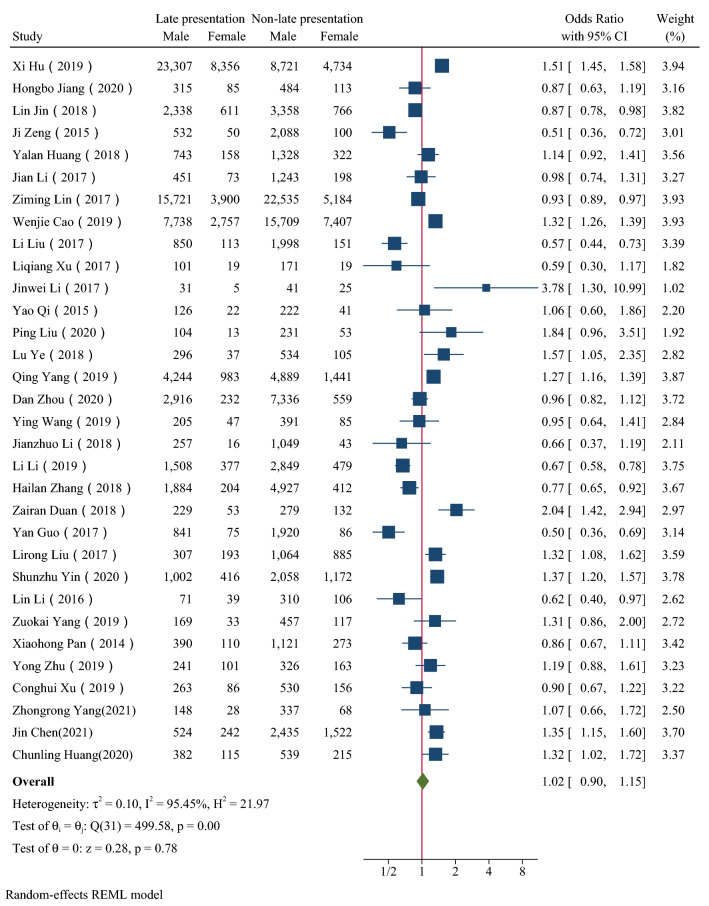
The forest plot of the association between gender and late presentation. The midpoint and length of each segment indicated the OR and 95% confidence interval. The diamond shape revealed the pooled OR

**Fig. 4:**
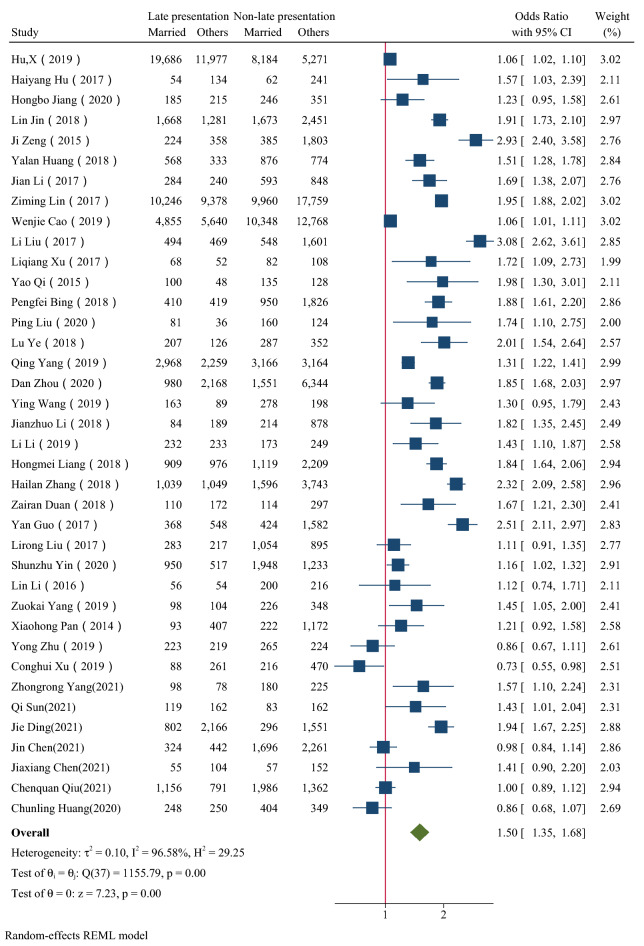
The forest plot of the association between marital status and late presentation. The midpoint and length of each segment indicated the OR and 95% confidence interval. The diamond shape revealed the pooled OR

**Fig. 5:**
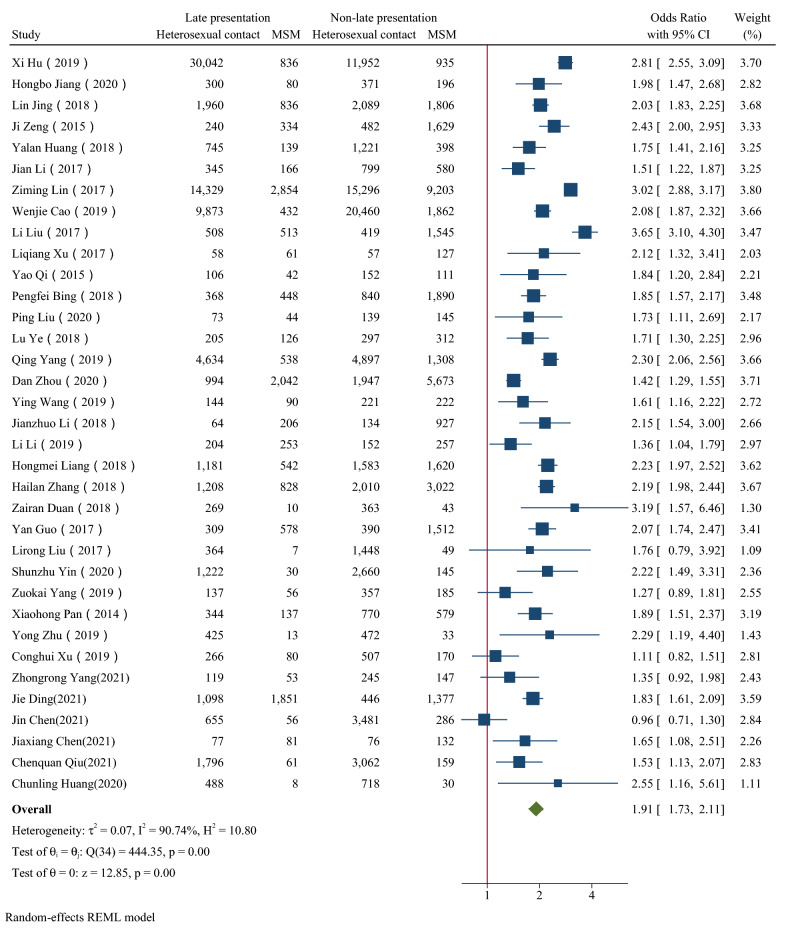
The forest plot of the association between risk factor for infection and late presentation. The midpoint and length of each segment indicated the OR and 95% confidence interval. The diamond shape revealed the pooled OR

Figure 2, 3, 4, 5, 6 showed the overall OR value of factors associated with LP. Generally, patients ≥ 50 years old (*OR* = 2.19, 95% CI: 1.85–2.58; *I*^*2*^ = 97.44%), married (*OR* = 1.50, 95% CI: 1.35–1.68; *I*^*2*^ = 96.58%), with heterosexual contact as risk factor for infection (*OR* = 1.91, 95% CI: 1.73–2.11; *I*^*2*^ = 90.74%) and diagnosed in medical institutions (*OR* = 2.35,95% CI: 2.11–2.62; *I*^*2*^ = 96.05%) were more likely to be diagnosed late. While male was not a risk factor for LP (*OR* = 1.02, 95% CI: 0.90–1.15; *I*^*2*^ = 95.45%). In high prevalence areas, patients ≥ 50 years old (*P* = 0.01), married (*P* < 0.01) and diagnosed in medical institutions (*P* = 0.01) were less likely to be presented late than in middle or low areas.

**Fig. 6:**
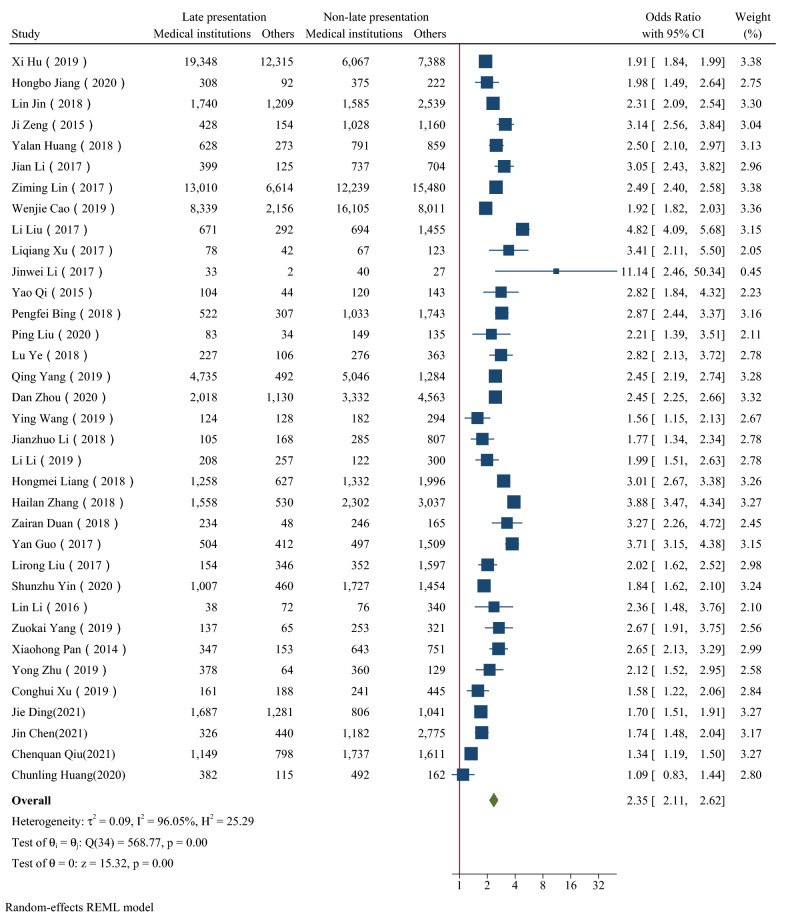
The forest plot of the association between sample sources and late presentation. The midpoint and length of each segment indicated the OR and 95% confidence interval. The diamond shape revealed the pooled OR

### Subgroups analyses by study regions and time

As shown in Table 2, in high prevalence regions, the proportion of LP patients < 50 years old, ≥ 50 years old, male, female, married and other marital status, infected by MSM, diagnosed in medical facilities and other institutions were all higher than middle or low prevalence regions (*P* < 0.01). For patients < 50 years old, ≥ 50 years old, male, female, married, with heterosexual contact as risk factor for infection, diagnosed in medical facilities and other institutions, the LP ratio decreased in 2016–2020 compare to 2010–2015 (*P* < 0.01). While in middle or low epidemic areas, the proportion of LP during 2016–2020 was high than before, and the difference was statistically significant (*P* = 0.015). As presented in Table 3. It is displayed in Table 4 that in high epidemic areas, patients ≥ 50 years old (*OR* = 1.67, 95% CI:1.35—2.07; *I*^*2*^ = 87.85%; *P* = 0.01), male (*OR* = 1.22, 95% CI:1.00—1.48; *I*^*2*^ = 85.94%; *P* = 0.04), married (*OR* = 1.18, 95% CI:1.00—1.38; *I*^*2*^ = 91.57%; *P* < 0.01) and diagnosed in medical institutions (*OR* = 1.89, 95% CI:1.58—2.26; *I*^*2*^ = 84.38%; *P* = 0.01) were less likely to be presented late than in middle or low areas. There were no statistically significant group differences. The risk of LP from 2020 to 2015 was 1.81, (95% CI: 1.45–2.26; *I*^*2*^ = 91.57%), 3.00, (95% CI: 2.52–3.58; *I*^*2*^ = 84.38%) respectively for patients married and diagnosed in medical institutions, whereas from 2016 to 2020 was 0.94, (95% CI: 0.78–1.14; *I*^*2*^ = 65.12%), 1.57, (95% CI: 1.24–1.99; *I*^*2*^ = 74.55%). The observed differences in risk estimates between the groups were statistically significant (*P* < 0.01).

**Table 2:** The overall proportion of LP of subgroups with different characteristics

		High prevalence regions n (LP%))	Middle or low prevalence regions n (LP%)	*P*-value	2010–2015 n (LP%)	2016–2020 n (LP%)	*P*-value
Age	≥ 50	23,968(66.02%)	7390(54.07%)	< 0.01*	748 (46.52%)	599 (33.92%)	< 0.01*
	< 50	32,681(43.60%)	13,612(31.93%)	< 0.01*	2399 (25.28%)	916 (18.36%)	< 0.01*
Gender	Male	41,858(52.14%)	26,376(33.21%)	< 0.01*	6038 (30.85%)	1484 (27.12%)	< 0.01*
	Female	13,399(48.79%)	6150(32.00%)	< 0.01*	1310 (33.08%)	528 (20.84%)	< 0.01*
Marital status	Married	33,244 (56.31%)	17,332 (39.85%)	< 0.01*	3692 (40.71%)	845 (24.80%)	< 0.01*
	Others	24,013 (44.46%)	20,148 (30.60%)	< 0.01*	3808 (25.64%)	1168(25.40%)	0.743
Infection routes	Heterosexual transmission	49,465 (55.69%)	25,685 (38.42%)	0.627	4234(39.06%)	1709(25.18%)	< 0.01*
	MSM	3942 (26.30%)	10,489 (27.56%)	< 0.01*	2674(25.20%)	224(24.72%)	0.752
Sample sources	Medical institutions	35,956 (59.48%)	26,472 (41.04%)	< 0.01*	4496(43.50%)	1177(33.95%)	< 0.01*
	Others	21,300 (40.58%)	10,239 (23.28%)	< 0.01*	2851 (21.60%)	835 (18.81%)	< 0.01*
Overall		57,266(50.66%)	43,041(36.21%)	< 0.01*	7615(31.70%)	2012(25.13)	< 0.01*

^*^Refers to a statistically significant difference (*P* < 0.05)

**Table 3:** The overall proportion of different time period in high epidemic areas and middle or low epidemic areas

Study region	Time period	Late presentation	Non Late presentation	*P*-value
High prevalence regions	2010–2015	720(22.82%)	2435(77.18%)	0.104
	2016–2020	1747(24.3%)	5442(75.70%)	
middle or low prevalence regions	2010–2015	7005(33.28%)	14,045(66.72%)	0.015*
	2016–2020	660(36.07%)	1170(63.93%)	

^*^Refers to a statistically significant difference (*P* < 0.05)

**Table 4:** The results of subgroup meta-analysis by study regions and time period

	Pooled OR (95%CI)		*P*-value	Pooled OR (95%CI)		*P*-value	Publication bias
	High prevalence regions	Middle or low prevalence regions		2010–2015	2016–2020		*P*-value
Age	1.67 (1.35–2.07)	2.50 (2.04–3.05)	0.01*	2.48 (1.77–3.49)	1.79 (1.43–2.25)	0.12	0.4632
Gender	1.22 (1.00–1.48)	0.94 (0.81–1.08)	0.04*	0.80 (0.63–1.02)	1.11 (0.88–1.41)	0.06	0.6793
Marital status	1.18 (1.00–1.38)	1.65 (1.46–1.87)	< 0.01*	1.81 (1.45–2.26)	0.94 (0.78–1.14)	< 0.01*	0.814
Infection routes	2.08 (1.57–2.74)	1.87 (1.70–2.06)	0.49	2.13 (1.77-2.57)	1.43( 0.94–2.17)	0.09	0.1438
Sample sources	1.89 (1.58–2.26)	2.57 (2.28–2.89)	0.01*	3.00 (2.52–3.58)	1.57 (1.24–1.99)	< 0.01*	0.3912

^*^Refers to a statistically significant difference (*P* < 0.05)

### The quality of evidence

The quality grade of age, infection routes, and sample sources were high. The grade score of gender and marital status was moderate. An additional file shows this in more detail (see Additional file 1).

### Sensitivity analysis

A leave-one-out sensitivity analysis was adopted to examine the possible cause of heterogeneity across the studies involved in the analysis. The sensitivity analysis results suggested that none of the individual studies influenced the initial total results.

### Publication bias

The conventional funnel plots indicated showed almost no publication bias in the meta-analysis. An additional file shows this in more detail (see Additional file 2). We used Harbord-test to confirm the result and found no statistically significant differences (*P* > 0.05) (Table 4).

## Discussion

We conducted 39 publications to identify the related factors of LP in China. It showed that the overall LP proportion from 2010 to 2020 in China was 43.26%. Patients ≥ 50 years old, married, with heterosexual contact as risk factor for infection, and diagnosed in medical institutions were risk factors of LP. Gender had no statistically significant relationship with LP. In high prevalence areas, patients who were ≥ 50 years old, married, and diagnosed in medical institutions were less likely to be presented late than in other areas. It suggested the need for targeted measures to reduce the occurrence of LP in different regions. Additionally, we have made some suggestions on prevention and policy making of LP to HIV care based on these data.

In the general health environment, the elderly and female should be associated with reduced odds of LP because they have better health seeking behaviours. However, in our study the results were different. In China, partly of the HIV positive female did not realize the risk because they were infected by their husband. That might lead to the result after pooling the articles. Studies have shown that the elderly have limited access and ability to obtain and understand HIV/AIDS prevention information [57–59]. Additionally, the elderly tend to ignore HIV infection due to various comorbidity symptoms [60]. Action plan for AIDS containment and prevention in 13th Five-Year Plan in China proposed to improve the pertinence of publicity and education. But the risk of LP by the elderly did not decrease. For different age groups, we need to carry out targeted publicity and education activities. For the elderly, we need to invest even more energy to conduct these activities. Married patients have weak awareness of HIV counseling and testing. That may be due to the influence of family life, discrimination sensitivity, and other factors. Many of them didn't present until the diagnosis of their spouses. Therefore, policies that seek to protect a spouse's right to be notified on time of HIV infection should be encouraged. At present, the main route of HIV infection is sexual contact [61]. Our study found that patients infected by heterosexual contact had a higher occurrence of LP compared with MSM. Prominently, some HIV patients got infected through extramarital and commercial sex. They probably did not get tested in time because of the fear of HIV stigma. Social support is particularly crucial for high-risk groups to teat actively and timely. LP patients are more likely to be found in medical institutions, such as STD clinics and pre-surgery. Hence the current work of consulting and testing still needs improvement. For this part of patients, we believe that enriching the consulting and testing methods can effectively improve the poor situation, such as carrying out online consulting and starting high-sensitivity self-testing.

In subgroup analysis, the proportion of LP in high epidemic areas is higher than in middle or low regions. When comparing the occurrence of LP in two time periods in middle or low epidemic areas, the latter period was significantly higher than the former. Action plan for AIDS containment and prevention in China in 12th Five-Year put forward measures to expand the coverage of publicity and education, comprehensive intervention, testing and treatment [62]. Even though great efforts had done to expand the coverage of testing and treatment of HIV, LP is still a pressing problem in high prevalence regions. In recent years, the situation of LP in middle or low prevalence areas had become more severe than before. Thus, we should continue to expand the coverage of testing and treatment. The government should invest more funds in high prevalence regions to conduct focused testing or even census for high-risk groups to find more patients as early as possible. Besides, we recommend the exemption of additional tests in high LP areas. So that ART can start as soon as HIV positives are confirmed irrespective of national guidelines. In middle or low prevalence regions, people over 50 years old, married, and examined in medical institutions should become the focus of HIV education, counseling, and testing.

There are some other findings in this study. Firstly, previous studies on the influencing factors of LP to HIV care mainly focused on social demographic determinants. There are still many other related factors to be explored, such as behavioral factors, AIDS knowledge level, access to testing and ART, policy, and social support. Secondly, there are few studies on the ART and immune reconstitution of LP patients. There are several limitations. The criteria for LP for the included publications were different, and the results may deviate from the real world. Therefore, there is an urgent need for a consensus definition of LP to facilitate full use of the actual material to reflect the problems and find solutions. Secondly, we included four articles in the 2016–2020 group, which may have led to bias in our results.

Generally, LP remains an obstacle to the prevention and treatment of HIV/AIDS in China. Targeted public health interventions to improve early entry into HIV care are urgently needed. We still have a lot to do for HIV-related policy-making, testing strategy, and health education in the future.

## Conclusion

Patients ≥ 50 years old, married, with heterosexual contact as risk factor for infection, and diagnosed in medical institutions were risk factors of LP. Gender had no significant relationship with LP. Although the country have expanded the coverage of testing and treatment of HIV through great efforts, LP is still a pressing problem in high prevalence regions. In middle or low prevalence areas, patients who were ≥ 50 years old, married, and diagnosed in medical institutions were more likely to be presented late than in other areas. Patients married and diagnosed in medical institutions after 2015 have a lower risk of LP than before. Governments should also take measures to expand the coverage of education, testing, and treatment of HIV.

## Supplementary Information

**Additional file 1**. The quality grade of patients exposed to different factors with late presentation compare to non-late presentation.

**Additional file 2.** The conventional funnel plots of different factors.

## Data Availability

All data generated or analyzed during this study are included in this published article [and its supplementary information files].

## Abbreviations

HIV: Human immunodeficiency virus
AIDS: Acquired immunodeficiency syndrome
LP: Late presentation (hereon in referred to as LP) to HIV care
ART: Antiretroviral therapy
CNKI: China National Knowledge Infrastructure
NOS: Newcastle–Ottawa quality-assessment scale
GRADE: Grading of Recommendations Assessment, Development and Evaluation
MSM: Men who have sex with men
STD: Sexually transmitted diseases

## References

[1] Meléndez J, Reinhardt SW, O'Halloran JA (2019). Late presentation and missed opportunities for HIV diagnosis in Guatemala. AIDS Behav.

[2] Late Presentation Working Groups in EuroSIDA and COHERE (2020). Estimating the burden of HIV late presentation and its attributable morbidity and mortality across Europe 2010–2016. BMC Infect Dis.

[3] Jin X, Xiong R, Wang LY, Mao YR (2016). Analysis on the ' late diagnosis' (LD) phenomena among newly identified HIV/AIDS cases in China, 2010–2014. Zhonghua liu xing bing xue za zhi Zhonghua liuxingbingxue zazhi.

[4] Mocroft A, Lundgren JD, Sabin ML (2013). Risk factors and outcomes for late presentation for HIV-positive persons in Europe: results from the Collaboration of Observational HIV Epidemiological Research Europe Study (COHERE). PLoS Med.

[5] Krawczyk CS, Funkhouser E, Kilby JM (2006). Factors associated with delayed initiation of HIV medical care among infected persons attending a southern HIV/AIDS clinic. South Med J.

[6] Krentz HB, Auld MC, Gill MJ (2004). The high cost of medical care for patients who present late (CD4 <200 cells/microL) with HIV infection. HIV Med.

[7] Marks G, Crepaz N, Janssen RS (2006). Estimating sexual transmission of HIV from persons aware and unaware that they are infected with the virus in the USA. AIDS (London, England).

[8] Lv F (2017). Key strategy of the China action plan for the thirteen five-year plan for combating and prevention of AIDS. Chin J Dis Control Prevent.

[9] Wu ZY (2019). HIV/AIDS prevention strategy with Chinese characteristics. Chin J Dis Control Prevent.

[10] Johnson LF, Mossong J, Dorrington RE (2013). Life expectancies of South African adults starting antiretroviral treatment: collaborative analysis of cohort studies. PLoS Med.

[11] Jain V, Hartogensis W, Bacchetti P (2013). Antiretroviral therapy initiated within 6 months of HIV infection is associated with lower T-cell activation and smaller HIV reservoir size. J Infect Dis.

[12] Deeks SG, Lewin SR, Bekker LG (2017). The end of HIV: Still a very long way to go, but progress continues. PLoS Med.

[13] Martin GE, Frater J (2018). Post-treatment and spontaneous HIV control. Curr Opin HIV AIDS.

[14] Rodger AJ, Cambiano V, Bruun T (2019). Risk of HIV transmission through condomless sex in serodifferent gay couples with the HIV-positive partner taking suppressive antiretroviral therapy (PARTNER): final results of a multicentre, prospective, observational study. Lancet.

[15] Belay GM, Endalamaw A, Ayele AD (2019). Late presentation of HIV positive adults and its predictors to HIV/AIDS care in Ethiopia: a systematic review and meta-analysis. BMC Infect Dis.

[16] Gesesew HA, Tesfay Gebremedhin A, Demissie TD (2017). Significant association between perceived HIV related stigma and late presentation for HIV/AIDS care in low and middle-income countries: a systematic review and meta-analysis. PLoS ONE.

[17] Sun XS, Jing J, Zhang XH (2011). Marketization, population mobility, and HIV/AIDS——based on Guangdong province. Popul Dev.

[18] Hu X, Liang B, Zhou C (2019). HIV late presentation and advanced HIV disease among patients with newly diagnosed HIV/AIDS in Southwestern China: a large-scale cross-sectional study. AIDS Res Ther.

[19] Hu H, Yan H, Liu X (2017). Trends in late HIV diagnosis among men who have sex with men in Jiangsu province, China: results from four consecutive community-based surveys, 2011–2014. PLoS ONE.

[20] Jiang H, Liu J, Tan Z (2020). Prevalence of and factors associated with advanced HIV disease among newly diagnosed people living with HIV in Guangdong Province, China. J Int AIDS Soc.

[21] Jin L, Cheng XL, Qin YZ, Su B (2018). Analysis of the influential factors of late diagnosis among newly identified HIV/AIDS cases in Anhui Province, 2011–2015. Chin J Prev Med.

[22] Zeng J, Li Y, Ye JR (2015). Characteristics of late diagnosis cases of newly reported HIV/AIDS cases in Beijing, 2013. Chin J AIDS STD.

[23] Huang YL, Chen QT, Xu J (2018). Analysis on the late diagnosis of HIV/AIDS cases and its influencing factors in Quanzhou City, 2011–2017. Strait J Prev Med.

[24] Li J, Wang B, Yang CM, Wang XR (2017). Analysis of late diagnosis cases of newly reported HIV/AIDS cases in Gansu province (2013–2015). Chin J Viral Dis.

[25] Lin ZM, Li Y, Fu XB (2017). Characteristics and influencing factors for late-diagnosed HIV/AIDS cases in Guangdong Province, 2010–2016. South China J Prev Med.

[26] Cao W, Yuan Z, Yao Y (2019). Analysis of late diagnosis and its influencing factors of newly reported HIV/AIDS in Guizhou Province from 2014 to 2018. Chin J Dis Control Prev.

[27] Liu L, Zhu Z, Xu Y (2017). Analysis on the influencing factors of late diagnosis of newly identified HIV/AIDS cases in Nanjing from 2011 to 2015. Chin J Dis Control Prev.

[28] Xu L, Gu Y, Zhou C (2017). Proportion and factors of late diagnosis of the reported HIV/AIDS cases during 2010–2015 in Changshu county. Chin J Aids STD.

[29] Li JW, Shao T (2017). Influencing factors of late diagnosis of patients infected with HIV in Jingjiang city. Jiangsu J Prev Med.

[30] Qi Y, Cui Q (2015). Analysis of epidemiological characteristics of HIV/AIDS and influencing factors of late diagnosis in Yancheng. Jiangsu J Prev Med.

[31] Bing PF, Zhao YQ, Zhao XP (2018). Late detection of HIV infection and its influencing factors in Suzhou, 2012–2017. Jiangsu J Prev Med.

[32] Liu P, Wu J, Yao MF (2020). Analysis on the late detection of HIV infection cases in Zhangjiagang City, Jiangsu Province from 2013 to 2018. J Med Pest Control.

[33] Ye L, Zhang MH, Hu CF (2018). Characteristics and influencing factors of HIV / AIDS late detection cases in Zhenjiang City from 2010 to 2017. Jiangsu J Prev Med.

[34] Yang Q, Luo YL, Hu Q (2019). Late diagnosis of newly identified HIV/AIDS cases and influencing factors in Jiangxi province during 2014–2018. Chin J AIDS STD.

[35] Zhou D, Pan S, Gai X (2020). Analysis on the influencing factors of late diagnosis of newly identified HIV/AIDS cases in Liaoning Province, China, 2015–2018. Chin J Dermatovenereol.

[36] Wang Y, Zhang RH, Huang KP (2019). Analysis on characteristics and influencing factors of newly reported late-diagnosed HIV/AIDS cases, Heze city 2014–2018. Prev Med Tribune.

[37] Li J, Ren Q, Li X (2018). Late diagnosis of HIV infection and its influencing factors in Jinan in 2011–2016. China Prev Med.

[38] Li L, Wang H (2019). Analysis of the first CD4+T test of newly reported HIV/AIDS cases living in Linyi city from 2012 to 2017. Chinese J AIDS STD.

[39] Liang H, Nie X, Mu S (2018). Late diagnosis of newly identified HIV/AIDS cases and its influencing factors in Shanxi, 2011–2016. Chin J Aids STD.

[40] Zhang HL, Wei XL, Zhao X (2018). Influencing factors of late diagnosis of newly identified HIV/AIDS cases in Xi'an, 2011–2017. China Trop Med.

[41] Duan Z, Wang J, Li B (2018). Characteristics and influencing factors of late detected HIV/AIDS cases between 2012 and 2016 in Hejiang County of Sichuan Province. Chin J Dermatovenereol.

[42] Guo Y, Ning TL, Zhou N (2017). The characteristics of late-diagnosed HIV/AIDS cases in Tianjin from 2011 to 2015 and its influencing factors. Chin J Hum Sex.

[43] Liu LR, Wang XM (2017). Late detection and influencing factors of newly reported HIV/AIDS patients in Yining City, Xinjiang, 2010–2015. Chin J AIDS STD.

[44] Yin SZ, Huang LH, Yang LF (2020). Delayed HIV/AIDS diagnosis in Dali Bai Autonomous Prefecture from 2012 to 2018 and its influencing factors. Acad J Chin PLA Med School.

[45] Li L, Yang YL, Cao YF (2016). Proportion and influencing factors of delay on HIV diagnosis among newly reported HIV-infected Myanmar patients in Dehong area of Yunnan Province in 2015. Chin J Dis Control Prev.

[46] Yang ZK, Wang H, Fang YR (2019). Characteristics of late detection of newly reported HIV/AIDS cases in Shaoxing City. Prev Med.

[47] Pan XY, Chen L, Xu Y (2014). Characteristics and influencing factors of HIV / AIDS patients in Zhejiang Province in 2012. Chin J Prev Med.

[48] Zhu Y, Ma F, Wu W (2019). Analysis on the characteristics of delayed diagnosis of HIV/AIDS cases in Rongchang district of Chongqing from 2012 to 2017. Chin J Aids STD.

[49] Xu CH (2019). Late presentation of HIV / AIDS and its influencing factors in Chongqing.

[50] Yang ZR, Li J, Jin MH (2021). Delayed HIV diagnosis and its associated factors in Huzhou. Prev Med.

[51] Sun Q, Xu L, Zhang HJ (2021). Analysis of the first CD4 cell count test for newly detected HIV/AIDS cases in Weihai. Chin J AIDS STD.

[52] Ding J, Yan H, Wu S (2021). Related factors of first CD4 cell count test for newly detected HIV/AIDS cases in Wuhan from 2010 to 2018. Prev Med.

[53] Chen J, Ni MJ (2021). Analysis on epidemiological characteristics of late presentation HIV / AIDS cases in Xinjiang in 2019. Bull Dis Control Prev (China).

[54] Chen JX, Li BX, Zhang D (2021). Influencing factors of late diagnosis of new HIV/AIDS cases in Jimei District of Xiamen City, 2010–2019. J Community Med.

[55] Qiu CQ, Deng LY, Geng PC (2021). Factors influencing the late detection of HIV infection cases in Qujing from 2014 to 2019. Chin J AIDS STD.

[56] Huang CL (2020). Analysis of late detection of newly reported human immunodeficiency virus-infected people and acquired immune deficiency syndrome patients in Suining City of Sichuan Province. J Clin Med Pract.

[57] Jiang H, Xie N, Fan Y (2015). Risk factors for advanced HIV disease and late entry to HIV care: national 1994–2012 HIV surveillance data for Wuhan, China. AIDS Patient Care STDs.

[58] Abel T, Werner M (2003). HIV risk behavior of older persons. Eur J Pub Health.

[59] Winningham A, Corwin S, Moore C (2004). The changing age of HIV: sexual risk among older African American women living in rural communities. Prev Med.

[60] Althoff KN, Gebo KA, Gange SJ (2010). CD4 count at presentation for HIV care in the United States and Canada: are those over 50 years more likely to have a delayed presentation?. AIDS Res Ther.

[61] Lin H, Chen W, Xu Y (2015). Strategy of HIV/AIDS identification in a coastal prefecture in eastern China. Chin J Dis Control Prev.

[62] Notice of the General Office of the State Council on Printing and Distributing "12th Five-Year Plan" Action Plan to Contain and Prevent AIDS in China. Gazette of the State Council of the People's Republic of China, 2012(07): 7–14. (in Chinese)

