# Supplementary material for: HIV/AIDS late presentation and its associated factors in China from 2010 to 2020: a systematic review and meta-analysis
Source: AIDS Res Ther. 2021 Dec 11;18:96. doi: 10.1186/s12981-021-00415-2 (PMC8665516; doi:10.1186/s12981-021-00415-2)
Supplement: Supplementary file 1 — Additional file 1. The quality grade of patients exposed to different factors with late presentation compare to non-late presentation. [file 12981_2021_415_MOESM1_ESM.docx]

**Supplementary Table 1:** The quality grade of patients exposed to different factors with late presentation compare to non-late presentation

| **Certainty assessment** | | | | | | | **No of patients** | | **Effect** | **Certainty** | **Importance** |
| --- | --- | --- | --- | --- | --- | --- | --- | --- | --- | --- | --- |
| **No of studies** | **Study design** | **Risk of bias** | **Inconsistency** | **Indirectness** | **Imprecision** | **Other considerations** | **Case** | **Control** | **Relative (95% CI)** |  |  |
| **Age** | | | | | | | | | | | |
| 26 | observational studies | not serious | Serious ^1^ | not serious | not serious | strong association ^2^ | 77651 | 90899 | **2.19 (1.85 to 2.58)** | ⨁⨁⨁⨁  HIGH | CRITICAL |
| **Gender** | | | | | | | | | | | |
| 32 | observational studies | not serious | serious ^3^ | not serious | not serious | none ^4^ | 86515 | 115727 | **1.02 (0.90 to 1.15)** | ⨁⨁⨁◯  MODERATE | CRITICAL |
| **Marital status** | | | | | | | | | | | |
| 38 | observational studies | not serious | serious ^5^ | not serious | not serious | none ^4^ | 92789 | 129664 | **1.50 (1.35 to 1.68)** | ⨁⨁⨁◯  MODERATE | CRITICAL |
| **Infection routes** | | | | | | | | | | | |
| 35 | observational studies | not serious | serious ^6^ | not serious | not serious | strong association ^2^ | 94157 | 126683 | **1.91 (1.73 to 2.11)** | ⨁⨁⨁⨁  HIGH | CRITICAL |
| **Sample sources** | | | | | | | | | | | |
| 35 | observational studies | not serious | serious ^7^ | not serious | not serious | strong association ^2^ | 93968 | 126551 | **2.35 (2.11 to 2.62)** | ⨁⨁⨁⨁  HIGH | CRITICAL |

^1^ Inconsistency: Rated as Serious. There is unexplained heterogeneity with an I^2^ of 97.44%;

^2^ Imprecision: Rated as Serious. It is due to the effect is large (OR>2.0). There is no evidence of dose-response gradient;

^3^ Inconsistency: Rated as Serious. There is unexplained heterogeneity with an I^2^ of 94.45%;

^4^ Imprecision: There is no evidence of dose-response gradient;

^5^ Inconsistency: Rated as Serious. There is unexplained heterogeneity with an I^2^ of 94.20%;

^6^ Inconsistency: Rated as Serious. There is unexplained heterogeneity with an I^2^ of 90.74%;

^7^ Inconsistency: Rated as Serious. There is unexplained heterogeneity with an I^2^ of 92.59%.
