# Supplementary figures and images for: HIV/AIDS late presentation and its associated factors in China from 2010 to 2020: a systematic review and meta-analysis
Source: AIDS Res Ther. 2021 Dec 11;18:96. doi: 10.1186/s12981-021-00415-2 (PMC8665516; doi:10.1186/s12981-021-00415-2)

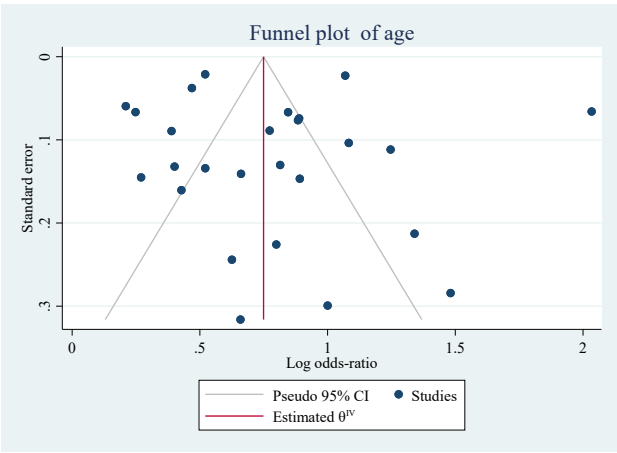

(a)

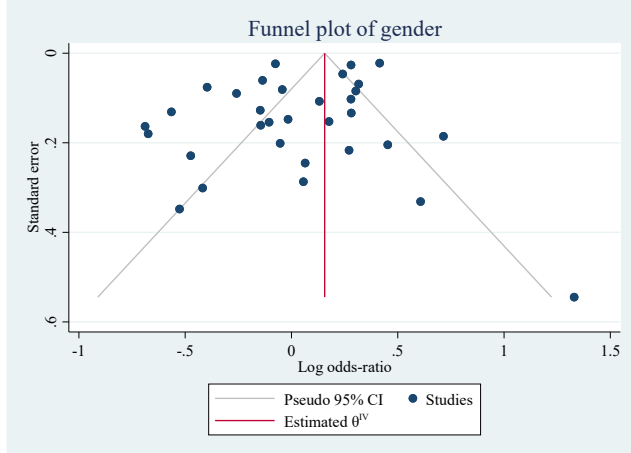

(b)

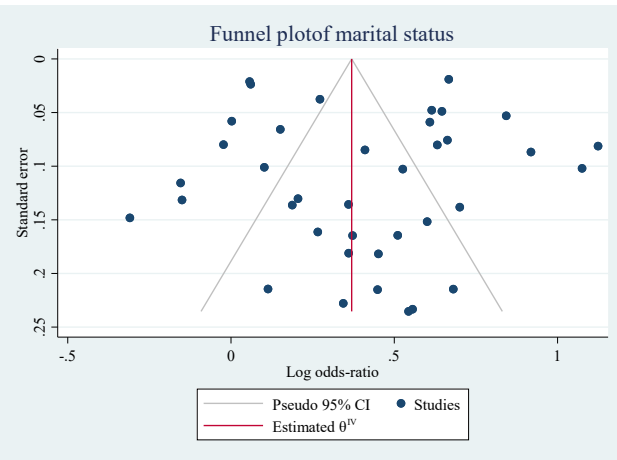

(c)

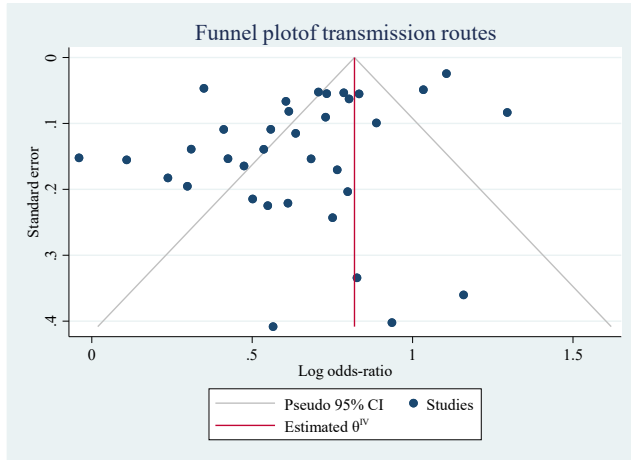

(d)

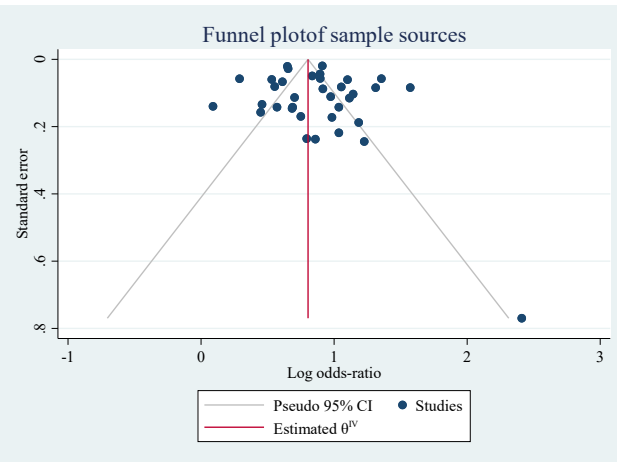

(e)

Supplement: Supplementary file 2 — Additional file 2. The conventional funnel plots of different factors. [file 12981_2021_415_MOESM2_ESM.pdf]
